# Zinc and copper status, biological aging, and mild cognitive impairment: cross-sectional mediation analysis in a high-risk occupational population

**DOI:** 10.1186/s12889-026-27057-1

**Published:** 2026-03-17

**Authors:** Kexin Cao, Yaqi Xu, Qian Gao, Limei Cao, Tong Wang

**Affiliations:** 1 Department of Health Statistics, School of Public Health, Shanxi Medical University, No. 56, Xinjian South Road, Taiyuan, Shanxi 030001 China; 2 Key Laboratory of Coal Environmental and Pathogenicity Prevention, Ministry of Education, Shanxi Medical University, Taiyuan, Shanxi China

**Keywords:** Zinc, Copper, Mild cognitive impairment, Biological age, Coal miners

## Abstract

**Background:**

Occupational exposures and long-term work-related stressors may accelerate biological aging and contribute to cognitive impairment among high-risk working populations. Zinc (Zn) and copper (Cu) are essential trace elements involved in neurophysiological regulation and may affect cognitive function during aging. However, it remains unclear whether biological age (BA) serves as a potential intermediary factor in the association between Zn and Cu status and mild cognitive impairment (MCI) among occupational populations.

**Methods:**

This study measured whole blood Zn and Cu levels among coal miners from a large-scale mining facility in northern Shanxi Province, China. Multivariable logistic regression was employed to examine the relationship of Zn and Cu concentrations with MCI. Potential dose-response patterns and non-linear trends were further evaluated using restricted cubic spline (RCS) functions. Constructed the BA to reflect the physiological status of coal miners by integrating multiple biochemical and functional indicators. In addition, mediation analyses were performed to quantify the indirect association of BA within these relationships.

**Results:**

Participants with moderate whole blood Zn levels exhibited a reduced likelihood of MCI (OR = 0.51, 95% CI: 0.31–0.86), whereas the association attenuated at higher concentrations. RCS analysis suggested a U-shaped pattern, with the lowest MCI risk observed at intermediate Zn levels. BA statistically explained 28.53% of the association between Zn and MCI in cross-sectional mediation analysis. Whole blood Cu levels did not show a significant relationship with the risk of MCI.

**Conclusion:**

These findings suggest a non-linear association between Zn status and cognitive health in occupational populations, with potential involvement of aging-related processes reflected by BA. Incorporating aging-related indicators into occupational health assessment may help identify workers at elevated risk of cognitive impairment.

**Supplementary Information:**

The online version contains supplementary material available at 10.1186/s12889-026-27057-1.

## Introduction

The global burden of cognitive impairment is escalating, posing significant challenges to healthcare systems worldwide. In China, approximately 44% of middle-aged and older individuals experience some degree of cognitive decline, and the prevalence increases markedly with advancing age [1]. Cognitive impairment commonly affects domains such as concentration, memory retention, and higher-order executive abilities, and can evolve from MCI toward more severe forms of dementia, including Alzheimer’s disease (AD), presenting a valuable opportunity for early recognition and management [2, 3]. While aging is the primary risk factor, emerging evidence suggests that occupational exposures can accelerate cognitive decline, placing certain high-risk working populations under additional threat [4–6]. Coal miners represent a particularly vulnerable group, especially in China, the world’s largest coal producer [7]. In addition to the established risk of pneumoconiosis, miners experience long-term exposure to various neurotoxic agents, including metallic elements and airborne particles, as well as occupational stressors like shift schedules and physical labor, which may collectively compromise cognitive performance [8–10]. Therefore, investigating the determinants of cognitive impairment in this large, understudied population is of critical public health importance.

Previous epidemiological and experimental studies have highlighted the significance of dietary trace elements, especially Zn and Cu, in neurocognitive function. Zn and Cu act as critical cofactors for numerous enzymes involved in synaptic transmission, neurodevelopment, and protection against oxidative stress [11–16]. Insufficient Zn levels have been linked to declines in cognitive performance and elevated oxidative stress, while high Cu exposure may induce neurotoxic effects via pathways involving oxidative damage [17–20]. Consequently, their homeostatic dysregulation has been implicated in the pathogenesis of cognitive decline. A major limitation of previous research is its predominant focus on the general elderly population, largely overlooking the context of chronic occupational exposure. For industrial workers like miners, exposure routes, such as inhalation of metal-containing dust, and nutritional status may differ markedly from the general population, potentially leading to distinct exposure-cognition relationships [21, 22]. Despite its public health importance, research in this occupational setting is scarce.

Biological age (BA), calculated from a combination of physiological and biochemical markers, is considered a more precise indicator of age-related physiological condition compared with chronological age (CA) [23]. Recent studies suggest that accelerated biological aging is associated with cognitive decline and neurodegenerative risks [24, 25]. Moreover, trace elements such as Zn and Cu may influence aging processes through oxidative stress, inflammation, and metabolic regulation [26, 27]. Nevertheless, limited research has explored the potential role of biological aging in the association between trace element levels and cognitive function, especially among workers exposed to chronic environmental hazards. To date, few studies have examined whether biological age may serve as a potential intermediary in the associations between Zn and Cu levels and MCI, particularly among coal miners. These gaps highlight the need for studies examining trace element status, biological aging, and cognitive function specifically in occupationally exposed populations.

Accordingly, this study investigated the relationships between whole blood Zn and Cu levels and MCI within a cohort of coal miners in northern China. In addition, we constructed a biological age index based on multiple biochemical and functional indicators and explored its potential role as a mediating factor in these associations. By investigating these associations among a high-risk occupational group, this study seeks to offer new epidemiological understanding of trace element levels, biological aging, and cognitive function, which could contribute to improved occupational health evaluation and preventive measures.

## Methods

### Study populations

This cross-sectional study utilized data from the 2023 TONGMEI survey, an occupational health monitoring program involving coal miners at a major mining company in northern Shanxi, China. Participant recruitment took place between January and March 2023 during an on-site health examination organized by the enterprise.

A two-stage proportionate stratified cluster sampling strategy was employed. First, 10 mining sites were randomly selected from a total of 87 mines distributed across three distinct mining areas (Pingwang, Kouquangou, and Yungangou). Second, workers within the selected mines were categorized into strata based on age, sex, and occupational role, and individuals were then randomly drawn from each stratum in proportion to the overall workforce distribution. Participants were eligible for inclusion if they: (1) were enrolled in the TONGMEI study; (2) were aged 18 years or older; (3) had complete whole blood Zn and Cu measurements; and (4) underwent a full cognitive assessment for MCI evaluation. Participants were not included in the analysis if information on Zn or Cu levels, or on cognitive assessment results, was unavailable. Following these criteria, a total of 585 participants were retained for the primary analyses. A comparison of baseline characteristics between included and excluded participants is provided in Table S1.

**Fig. 1:**
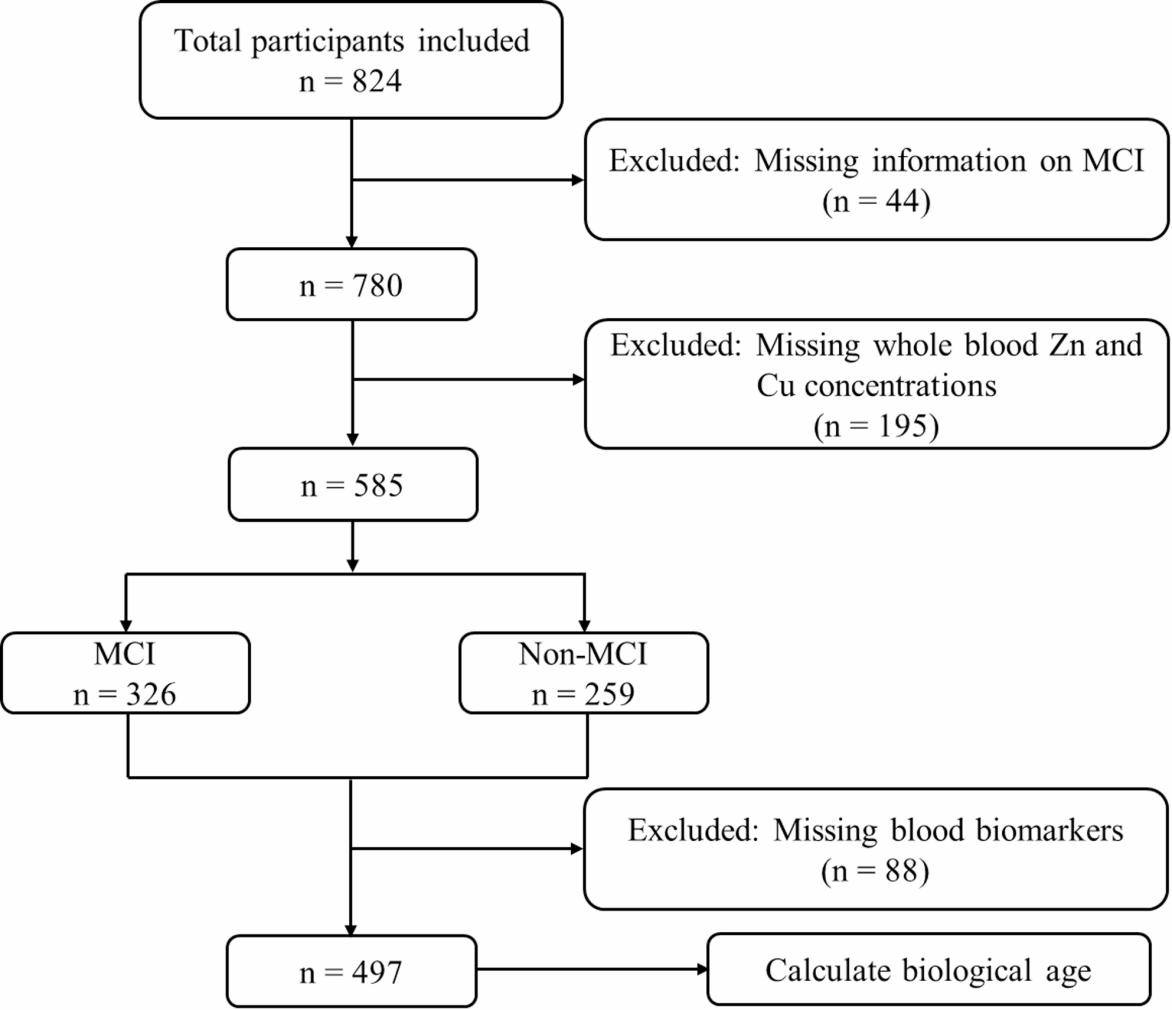
Participant screening and selection flowchart

For the analysis involving BA, participants who lacked data for any of the 56 biomarkers used to calculate BA were excluded, producing a final sample of 497 individuals specifically for mediation analysis. The detailed participant selection process is presented in Fig. 1.

### MCI assessment

The Montreal Cognitive Assessment (MoCA), developed by Dr. Nasreddine in Canada in 2005, is an evaluation instrument commonly used to detect MCI. It is informed by clinical practice and incorporates elements adapted from the Mini-Mental State Examination (MMSE) [28]. The MoCA assesses eight cognitive domains, covering attention and concentration, executive functioning, memory, language, visuospatial abilities, abstract reasoning, numerical calculation, and orientation. The instrument comprises 11 items, yielding a maximum score of 30, with scores of 26 or higher indicating normal cognitive performance. This study employed the Beijing version of the MoCA (MoCA-BJ), a culturally and linguistically adapted version of the original MoCA designed for the Chinese population. The MoCA-BJ has been tested in both urban and rural populations, showing high sensitivity (90.4%) and moderate specificity (31.3%) for identifying MCI [29]. Participants with a total score below 26 were classified as having MCI.

### Determination of Zn and Cu concentration

Whole blood levels of Zn and Cu were determined for all participants in this study. Following at least a 10-hour overnight fast, venous blood was drawn from the antecubital vein between 7:00 and 9:00 a.m. by trained nurses. Samples were collected into trace element-free vacuum tubes, gently mixed, and transported to the hospital laboratory under cold-chain conditions (4 °C) within 2 h of collection. Colorimetric assays (Beijing Jiuqiang Biotechnology Co., Ltd., China) were used to determine whole blood Zn and Cu levels, performed on an automated clinical chemistry analyzer (SIEMENS ADVIA 1800, Siemens Healthcare Diagnostics, Germany). The ADVIA 1800 is a fully automated chemistry analyzer capable of high-throughput colorimetric assays with multi-wavelength spectrophotometric detection.

For Zn, a 5-bromo-PAPS colorimetric method was used (LOD ≤ 0.1 µmol/L); for Cu, a Di-Br-PAESA colorimetric method was applied (LOD ≤ 0.5 µmol/L). All measured Zn and Cu concentrations in the samples were above the respective limits of detection. All analyses were conducted in a certified hospital clinical laboratory under standardized quality control procedures and in accordance with national laboratory standards.

The study adhered to the ethical guidelines outlined in the Declaration of Helsinki and received approval from the Shanxi Medical University Ethics Committee (No. 2023SJL35). All participants provided written informed consent before taking part in the study.

### Construction of BA

In this study, BA of coal miners was calculated using the Klemera-Doubal method (KDM), originally proposed by Klemera and Doubal [30]. The KDM estimates BA by integrating multiple biomarkers through a weighted regression framework that minimizes the discrepancy between predicted BA andCA. The BA for each individual was calculated using the formula:

$$\mathrm{B}\mathrm{A}=\frac{\sum\limits_{\mathrm{j}=1}^{\mathrm{m}}\left({\mathrm{x}}_{\mathrm{j}}-{\mathrm{q}}_{\mathrm{j}}\right)\left(\frac{{\mathrm{k}}_{\mathrm{j}}}{{\mathrm{s}}_{\mathrm{j}}^{2}}\right)+\frac{\mathrm{C}\mathrm{A}}{{\mathrm{s}}_{\mathrm{B}\mathrm{A}}^{2}}}{\sum\limits_{\mathrm{j}=1}^{\mathrm{m}}\left(\frac{{\mathrm{k}}_{\mathrm{j}}}{{\mathrm{s}}_{\mathrm{j}}^{2}}\right)+\frac{1}{{\mathrm{s}}_{\mathrm{B}\mathrm{A}}^{2}}}$$

Here, $j$ represents the indices for biomarkers and individual participants, respectively. The coefficients $k$, $q$, and $s$ and s represent the slope, intercept, and root mean square error (RMSE), respectively, calculated from the regression of each biomarker against CA.

First, we included 56 biomarkers and assessed their correlation with CA, retaining those with less than 30% missing data and an absolute correlation coefficient (|r|) greater than 0.1 with CA. The full list of the 56 candidate biomarkers and their correlation coefficients with CA is presented in Supplementary Table S3. Next, the inter-correlations among the candidate biomarkers were evaluated. For any pair of variables with a Pearson correlation coefficient |r| > 0.7, the more representative biomarker was selected based on clinical relevance and prior literature, prioritizing biomarkers with clearer physiological interpretability, wider use in aging research, and more independent biological information rather than derived indices. Fourteen biomarkers were ultimately incorporated into the KDM model, namely systolic blood pressure (SBP), forced vital capacity (FVC), alanine aminotransferase (ALT), high-density lipoprotein (HDL), monocyte percentage (MPNOP), red blood cells (RBC), lymphocytes (LYM), mean corpuscular volume (MCV), red blood cell distribution width-coefficient of variation (RBCCV), albumin (ALB), uric acid (UA), blood urea nitrogen (BUN), prothrombin time (PT), and activated partial thromboplastin time (APTTL).

### Covariates

In this study, trained personnel with medical backgrounds from Shanxi Medical University conducted face-to-face interviews with participants. The survey collected information on general demographic characteristics, dietary habits, and included standardized assessment scales. Covariates considered in the analysis comprised age, body mass index (BMI), sex, marital status (married, separated, widowed, divorced, never married), smoking status, alcohol consumption, educational level (Junior high school and below, Junior college and high school, Bachelor degree or above), monthly income (< 6,000 RMB, 6,000–10,000 RMB, ≥ 10,000 RMB), hypertension, and diabetes.

Adults who reported smoking at least one cigarette daily over the previous month were classified as smokers. Adults who reported drinking alcohol at least once a month over the previous year were classified as alcohol drinkers. Participants were considered hypertensive if they reported a physician diagnosis, were taking antihypertensive medications, or had a measured systolic blood pressure ≥ 130 mmHg and/or diastolic blood pressure ≥ 80 mmHg. Participants were classified as having diabetes if they reported a physician diagnosis, were using insulin or oral hypoglycemic medications, had a fasting blood glucose (FBG) ≥ 126 mg/dL, or a glycated hemoglobin (HbA1c) level ≥ 6.5%.

### Statistical analysis

To limit the impact of outliers, values below the 1st percentile and above the 99th percentile were winsorized by substituting them with the respective percentile thresholds. Continuous variables are expressed as mean ± standard deviation (SD), whereas categorical variables are reported as frequency and percentage (n, %). Differences in numerical and categorical variables between the MCI and non-MCI groups were assessed using independent samples t-tests and chi-square tests, respectively. Missing data in covariates were generally limited, ranging from 1.35% to 11.82% across variables (Table S2). Missing covariate values were handled using the MissForest algorithm, which is a non-parametric imputation method utilizing random forest models. This approach iteratively predicts missing values by leveraging information from other observed variables, and is capable of handling both continuous and categorical data with demonstrated reliability in epidemiological research [31]. All subsequent multivariable analyses were conducted on the dataset with imputed values.

To assess how Zn and Cu levels in whole blood relate to MCI risk, Zn and Cu concentrations were divided into quartiles. Both univariate and multivariate logistic regression analyses were performed, with the lowest quartile serving as the reference category. Model 1 included no covariates; Model 2 controlled for age and sex; and Model 3 additionally accounted for smoking, alcohol consumption, educational level, and monthly income. RCS regression was applied to explore possible nonlinear relationships between whole blood Zn and Cu levels and MCI. Knots were positioned at the 10th, 50th, and 90th percentiles of the Zn and Cu distributions. Covariates including age, sex, smoking, alcohol use, educational attainment, and monthly income were included in the model. Odds ratios (ORs) with 95% confidence intervals (CIs) were estimated using the median concentrations of Zn and Cu as reference values. All analyses were conducted using the “rms” package in R. To investigate whether BA might be involved in the relationship between Zn and Cu levels and MCI, a mediation analysis was performed using the “mediation” package in R with a bootstrap procedure (1,000 iterations), controlling for the covariates described above. Due to the cross-sectional nature of the study, the mediation analysis aimed to evaluate statistical indirect relationships rather than to draw causal inferences.

Statistical analyses were conducted using R software (version 4.4.1), and a two-tailed P value of less than 0.05 was regarded as statistically significant.

## Result

### Demographic characteristics of participants

A total of 326 participants (55.7%) out of the 585 included in the study were diagnosed with MCI. Table 1 presents the primary demographic and clinical features of the study participants. Of the total participants, 95.6% (*n* = 559) were men and 4.4% (*n* = 26) were women. Within the MCI subgroup, the mean age was 46.77 years, and males numbered 311, representing 95.4% of this group. Among those diagnosed with MCI, 37 individuals (11.3%) had diabetes, and 235 (72.1%) had hypertension.

**Table 1:** General demographic characteristics of participants

Variables	MCI (*N* = 326)	Non-MCI (*N* = 259)	*P*
Age,$\overline{\mathrm x}\pm\mathrm s$	46.77$\pm$ 6.14	43.73$\pm$5.99	**<0.001**
BMI,$\overline{\mathrm x}\pm\mathrm s$	25.45$\pm$3.44	25.51$\pm$3.44	0.844
Age group, n (%)			**<0.001**
≤ 45	127 (39.0)	160 (61.8)	
>45	199 (61.0)	99 (38.2)	
Gender, n (%)			0.996
Male	311 (95.4)	248 (95.8)	
Female	15 (4.6)	11 (4.2)	
Marital status, n (%)			0.721
Married	308 (94.5)	249 (96.1)	
Separated	2 (0.6)	0 (0.0)	
Widowed	10 (3.1)	6 (2.3)	
Divorced	2 (0.6)	1 (0.4)	
Never married	4 (1.2)	3 (1.2)	
Smoking, n (%)			0.725
No	105 (32.2)	79 (30.5)	
Yes	221 (67.8)	180 (69.5)	
Alcohol consumption, n (%)			**0.005**
No	205 (62.9)	192 (74.1)	
Yes	121 (37.1)	67 (25.9)	
Educational level, n (%)			**<0.001**
Junior high school and below	191 (58.6)	83 (32.0)	
Junior college and high school	11 (3.4)	5 (1.9)	
Bachelor degree or above	124 (38.0)	171 (66.0)	
Monthly income, (Yuan)			**<0.001**
<6,000	133 (40.8)	56 (21.6)	
6,000–10,000	150 (46.0)	146 (56.4)	
>10,000	43 (13.2)	57 (22.0)	
Diabetes, n (%)			0.244
No	289 (88.7)	238 (91.9)	
Yes	37 (11.3)	21 (8.1)	
Hypertension, n (%)			0.136
No	91 (27.9)	88 (34.0)	
Yes	235 (72.1)	171 (66.0)	
Score,$\overline{\mathrm x}\pm\mathrm s$	21.67$\pm$3.05	27.22$\pm$1.12	**<0.001**
BA,$\overline{\mathrm x}\pm\mathrm s$, (n)	49.08$\pm$24.88 (282)	40.74$\pm$22.50 (215)	**<0.001**
Zn (μmol/L),$\overline{\mathrm x}\pm\mathrm s$	16.82$\pm$2.91	17.30$\pm$2.68	**0.041**
Cu (μmol/L),$\overline{\mathrm x}\pm\mathrm s$	17.74$\pm$8.97	17.32$\pm$8.37	0.559

Statistically significant results (*p* < 0.05) are highlighted in bold

Relative to participants without MCI, those in the MCI group exhibited a higher frequency of alcohol use and lower educational attainment and monthly income (all *P* < 0.05). Furthermore, participants with MCI had a lower mean whole blood Zn concentration (16.82 ± 2.91 µmol/L) compared to those without MCI (17.30 ± 2.68 µmol/L, *P* < 0.05). By comparison, participants with MCI had a slightly higher mean Cu concentration (17.74 ± 8.97 µmol/L) than those without MCI, but this difference did not reach statistical significance (*P* > 0.05).

### Relationships between concentrations of Zn, Cu, and MCI

**Fig. 2:**
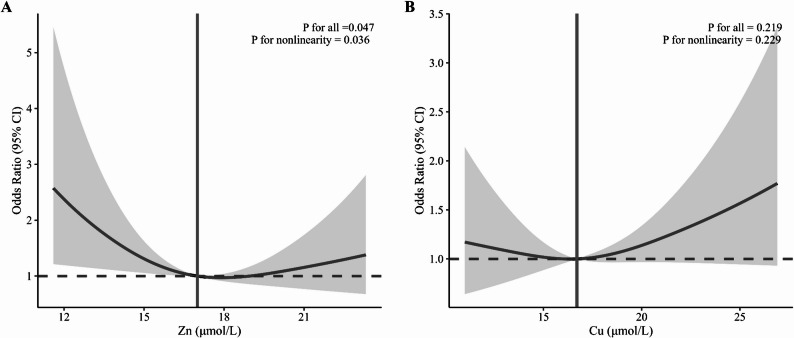
Dose-response relationships between whole blood Zn (**A**) and Cu (**B**) concentrations and mild cognitive impairment (MCI). Solid lines represent adjusted odds ratios (ORs), and shaded areas indicate 95% confidence intervals (95% CIs). The analysis included 585 participants (326 with MCI and 259 without MCI). All models were adjusted for age, gender, smoking, alcohol consumption, education level, and monthly income

Table 2 presents the relationships between whole blood Zn and Cu levels and the risk of MCI. In the unadjusted analysis, Zn treated as a continuous variable was inversely associated with the risk of MCI (OR = 0.94, 95% CI: 0.89–0.99). When examining Zn quartiles, participants in Q2 (OR = 0.45, 95% CI: 0.28–0.72) and Q3 (OR = 0.48, 95% CI: 0.30–0.77) had significantly lower odds of MCI compared to those in the lowest quartile (Q1), with a significant decreasing trend across quartiles (P for trend = 0.004). After adjusting for age, sex, smoking, alcohol consumption, educational level, and monthly income (Model 3), moderate Zn levels remained significantly associated with lower MCI risk in Q2 (OR = 0.49, 95% CI: 0.30–0.82) and Q3 (OR = 0.51, 95% CI: 0.31–0.86). In the highest quartile (Q4), the association weakened and did not reach statistical significance (OR = 0.73, 95% CI: 0.44–1.21); nevertheless, the overall trend across the quartiles remained statistically significant (P for trend = 0.023). RCS analysis revealed a U-shaped association between Zn levels and MCI risk (P for nonlinearity = 0.036), with the lowest odds of MCI seen at moderate Zn concentrations and a diminishing effect at higher levels (Fig. 2). 

**Table 2:** Associations of whole blood zinc (Zn) and copper (Cu) quartiles with mild cognitive impairment (MCI): odds ratios (ORs) and 95% confidence intervals (CIs)

Metal	Variables	Continuous	Quartile 1	Quartile 2	Quartile 3	Quartile 4	*P* for trend
Zn	MCI/non-MCI (n)	326/259	101/48	72/76	71/71	82/64	**-**
	Model 1	**0.94 (0.89**,**0.99)**	1(Ref.)	**0.45 (0.28**,**0.72)**	**0.48 (0.30**,**0.77)**	**0.61 (0.38**,**0.98)**	**0.004**
	Model 2	0.96 (0.90,1.01)	1(Ref.)	**0.45 (0.28**,**0.73)**	**0.52 (0.32**,**0.84)**	0.68 (0.41,1.10)	**0.008**
	Model 3	0.96 (0.90,1.03)	1(Ref.)	**0.49 (0.30**,**0.82)**	**0.51 (0.31**,**0.86)**	0.73 (0.44,1.21)	**0.023**
Cu	MCI/non-MCI (n)	326/259	85/61	72/74	83/63	86/61	-
	Model 1	1.01 (0.99,1.03)	1(Ref.)	0.70 (0.44,1.11)	0.95 (0.59,1.50)	1.01 (0.64,1.61)	0.345
	Model 2	1.01 (0.99,1.03)	1(Ref.)	0.67 (0.42,1.08)	0.90 (0.56,1.45)	0.96 (0.59,1.55)	0.344
	Model 3	1.01 (0.99,1.03)	1(Ref.)	0.70 (0.42,1.17)	0.91 (0.55,1.51)	1.03 (0.62,1.72)	0.423

The analysis included 585 participants (326 with MCI and 259 without MCI)

Model 1 represents the unadjusted analysis

Model 2 accounts for age and sex

Model 3 adjusts for age, gender, smoking status, alcohol use, educational attainment, and monthly income

Statistically significant results (*p* < 0.05) are highlighted in bold

By comparison, Cu levels were not significantly related to MCI risk in either the logistic regression or RCS analyses.

### Construction of BA

Among the 56 candidate biomarkers, 16 showed correlation coefficients greater than 0.1 with CA (Table S3). Several biomarkers were highly intercorrelated (|r| > 0.7, Figure S1), and representative variables were retained based on clinical relevance and prior literature, resulting in 14 biomarkers used to construct the BA model. The calculated BA showed a moderate-to-strong correlation with CA (*r* = 0.72, *p* < 0.001), suggesting that the derived BA effectively captures age-related physiological changes. Participants with MCI exhibited a higher mean BA than those without MCI (49.08 ± 24.88 vs. 40.74 ± 22.50 years, *P* < 0.001).

### Mediation analyses

We further explored whether BA statistically accounted for part of the association between Zn and MCI. Due to missing data on the 14 selected biomarkers in 88 out of 585 participants, the mediation analysis was conducted on the remaining 497 participants with complete data.

**Fig. 3:**
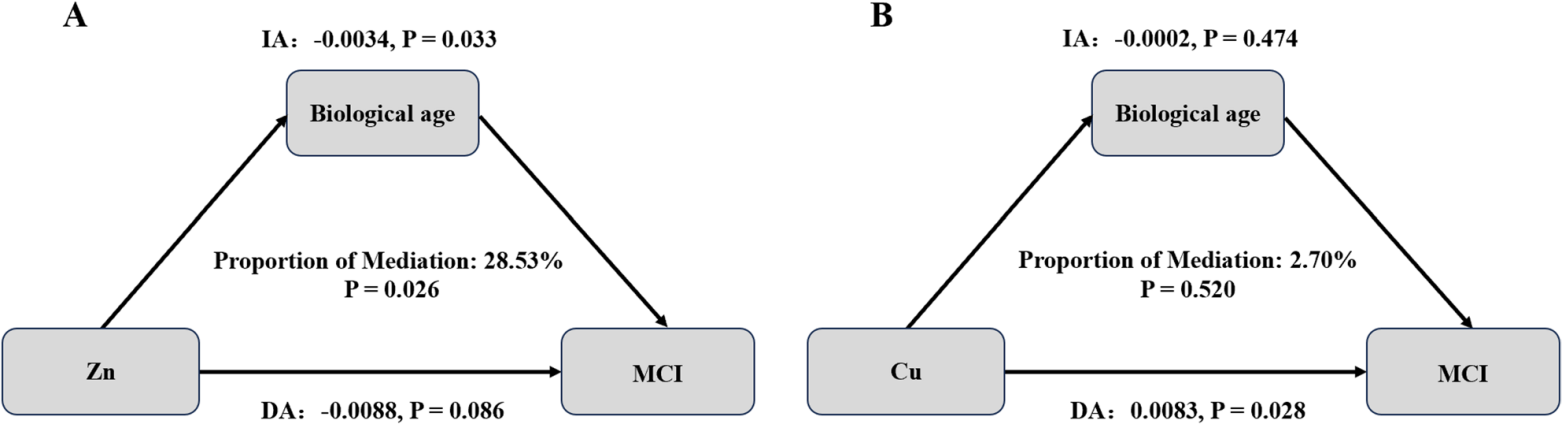
Statistical mediation model showing the associations between whole blood Zn (**A**) and Cu (**B**), biological age (BA), and mild cognitive impairment (MCI) (*N* = 497). Path coefficients representing direct and indirect associations are presented with corresponding P values. The proportion of the total association statistically attributed to BA is also shown. Abbreviations: DA, direct association; IA, indirect association; BA, biological age

As shown in Fig. 3, BA plays a partial mediation function in the connection between Zn and MCI. The estimated indirect association of Zn via BA was − 0.0034 (*P* = 0.033), contributing 28.53% to the total association (*P* = 0.026). The direct association was − 0.0088 and did not achieve statistical significance (*P* = 0.086). By contrast, no statistically significant association involving BA was found for Cu.

### The association between Zn, Cu, and MCI risk stratified by age

Given the potential impact of age on MCI risk, we performed a subgroup analysis by age to investigate the relationships between whole blood Zn and Cu levels and MCI. The results are summarized in Table S4-S5 and Figure S2. Among participants aged ≤ 45 years (*n* = 287), no statistically significant associations were detected between whole blood Zn or Cu concentrations and MCI risk in any model (all P for trend > 0.05). In Model 3, relative to the lowest quartile, the odds ratios for MCI in Q2, Q3, and Q4 were 0.51 (95% CI: 0.25–1.07), 0.53 (95% CI: 0.26–1.09), and 0.92 (95% CI: 0.46–1.84), respectively (P for trend = 0.143).

By contrast, in participants aged > 45 years (*n* = 298), a significant inverse relationship between Zn and MCI risk was detected in both the unadjusted and partially adjusted models. In Model 2, higher Zn concentrations as a continuous variable were associated with lower risk of MCI (OR = 0.90, 95% CI: 0.82–0.97). Relative to the lowest quartile, moderate to high Zn levels showed consistently reduced odds of MCI (Q2: OR = 0.38, 95% CI: 0.19–0.76; Q3: OR = 0.44, 95% CI: 0.21–0.92; Q4: OR = 0.40, 95% CI: 0.20–0.83; P for trend = 0.031). After full adjustment in Model 3, the association weakened, with only Q2 remaining significant (OR = 0.45, 95% CI: 0.21–0.96), and the trend across quartiles did not retain significance (P for trend = 0.199). RCS analyses further illustrated these associations (Figures S2).

## Discussion

The present study systematically investigated the associations of whole blood Zn and Cu levels with MCI among coal miners in northern Shanxi Province, and, for the first time, incorporated BA into the analytical framework to explore its potential intermediary role. Two key findings emerged. Whole blood Zn concentrations showed a non-linear association with MCI, with lower odds observed at moderate levels and attenuation at higher concentrations, suggesting a U-shaped pattern. Second, cross-sectional mediation analysis suggested that BA statistically accounted for 28.53% of the observed association between Zn and MCI, indicating that aging-related physiological changes reflected by BA may partly explain this relationship. In contrast, no significant relationship was observed between Cu levels and MCI risk.

Zn is primarily obtained through dietary intake and predominantly exists intracellularly in a bound form with metallothionein [32, 33]. In the brain, Zn homeostasis is essential for maintaining synaptic signaling pathways that underlie cognitive function, particularly through the modulation of NMDA receptor activity and LTP [34]. Consistent with these neurobiological roles, our findings indicated that moderate Zn concentrations were linked to reduced odds of MCI, whereas this relationship weakened at higher concentrations, highlighting the importance of maintaining optimal Zn levels for cognitive health. RCS analysis revealed a U-shaped pattern linking Zn concentrations with MCI probability, with the lowest likelihood observed in the second and third quartiles and a weakened association in the highest quartile. Experimental studies have demonstrated a dual role of Zn in synaptic plasticity: chelation of Zn disrupts LTP and impairs memory formation, whereas excessive Zn suppresses NMDA receptor function and inhibits long-term depression (LTD) [35]. This suggests that both Zn deficiency and overload are detrimental, disturbing the optimal balance required for hippocampal function and memory encoding. Therefore, imbalance in systemic Zn levels may disrupt this fine-tuned equilibrium, leading to suboptimal synaptic conditions for cognitive processing. The neuroprotective role of Zn may be particularly crucial in this occupational context, as coal miners face chronic exposures that can accelerate oxidative stress and biological aging [36, 37], processes against which Zn’s antioxidant and anti-inflammatory properties are thought to confer protection [38]. Moreover, the inverse association between Zn and MCI was more pronounced among individuals aged ≥ 45 years. This age-specific pattern may reflect the progressive decline of Zn status during the aging process, along with greater vulnerability of synaptic plasticity under conditions of Zn imbalance in midlife and later life [39].

In contrast, whole blood Cu concentrations tended to be higher in individuals with MCI compared with participants without MCI, although this variation did not reach statistical significance. While previous studies have reported elevated Cu levels in cognitively impaired populations [40, 41], our findings did not demonstrate a clear association between Cu concentrations and MCI in this occupational cohort. In the brain, Cu acts as a redox-active cofactor that supports antioxidant defense and interacts with endogenous molecules such as melatonin to regulate oxidative balance and circadian rhythm [42]. However, excessive Cu can engage in Fenton-type redox reactions that produce highly reactive hydroxyl species, thereby promoting oxidative stress, impairing mitochondrial function, and triggering neuroinflammatory processes, ultimately accelerating damage to nerve cells and cognitive decline [43]. Elevated Cu concentrations have been linked to reductions in working memory and higher-order cognitive processes in healthy adults, as well as an increased risk of AD [44, 45]. Although coal miners in our study exhibited relatively elevated Cu levels compared with reference populations reported in prior literature [46], the absence of a statistically significant association suggests that the role of Cu in cognitive impairment may be weaker or context-dependent in this group. It is also possible that compensatory antioxidant mechanisms mitigate Cu-related oxidative stress within this occupational population.

Lower Zn levels were linked more closely to cognitive deficits among the older participants of our cohort. Consistent with previous studies, age was positively associated with MCI and negatively correlated with whole blood Zn concentrations, likely reflecting age-related declines in Zn status due to reduced intake, impaired absorption, and increased vulnerability of synaptic plasticity [47]. Midlife and later-life cognitive changes, such as slower processing speed and hippocampal synaptic dysregulation, may interact with Zn deficiency to exacerbate cognitive decline [48, 49]. The results indicate that disturbances in Zn homeostasis could represent a key mechanism contributing to age-related cognitive decline, consistent with our observations in coal miners.

As an integrated measure of multiple physiological indicators, BA provides a more precise assessment of overall physiological status and age-related decline than CA [50, 51]. In this study, BA showed a potential mediating role in the association between whole blood Zn levels and MCI, accounting for 28.53% of the total association, indicating that aging-related physiological processes captured by BA may partly underlie the link between Zn status and cognitive function. Previous studies have indicated that Zn plays a role in regulating oxidative stress, supporting mitochondrial function, and modulating metallothionein-related inflammatory pathways, biological processes closely associated with aging and neurodegeneration [52, 53]. Considering that coal miners are chronically exposed to occupational stressors that may accelerate physiological aging, adequate Zn status may be associated with more favorable aging-related profiles and better cognitive performance in this population. However, because this study is cross-sectional, the results should be considered as statistical associations rather than proof of causal mediation.

This study represents the first investigation of the relationships among Zn, Cu, BA, and MCI in a cohort of coal miners, and it innovatively incorporates BA to explore potential pathways linking metal exposure to cognitive impairment. However, a number of study limitations merit consideration. To begin, roughly 29% of the cohort were excluded due to missing cognitive assessment or Zn/Cu measurements, primarily caused by incomplete MoCA responses, insufficient blood volume, or laboratory issues. Baseline comparisons indicated that included and excluded participants were largely comparable, although differences in gender, education, and income were observed and were adjusted for in multivariable models. While residual selection bias cannot be fully ruled out, the study sample is likely representative of the target population. Second, due to the cross-sectional design, Zn levels, biological age, and MCI were measured simultaneously, which precludes establishing temporal sequence among exposure, mediator, and outcome. Therefore, the observed role of BA should be understood as a statistical breakdown of associations rather than as proof of causal mediation. In addition, reverse causation cannot be ruled out, as cognitive impairment or underlying health conditions may also influence trace element status and aging-related biomarkers. Moreover, mediation analysis depends on the key assumption of sequential ignorability, meaning that there is no unmeasured confounding between the mediator and the outcome. Given the observational nature of our data, this assumption cannot be fully verified, and the possibility of residual confounding due to unmeasured variables cannot be completely ruled out. Accordingly, the mediating role of biological age should be interpreted cautiously as indicative of a potential associative pathway rather than definitive causal evidence. Third, the study sample was restricted to coal miners from a specific region in northern Shanxi, with a relatively small sample size and a markedly imbalanced sex ratio, potentially constraining the broader applicability of the results. Fourth, cognitive status was assessed using the MoCA-BJ, a screening rather than a diagnostic tool for cognitive impairment, which could lead to misclassification, particularly among individuals with lower educational attainment. Finally, although adjustments were made for multiple covariates, the possibility of residual confounding from unassessed factors, including dietary patterns, environmental exposures, or genetic susceptibility, cannot be entirely dismissed. Future longitudinal studies including larger and more heterogeneous populations are needed to confirm these findings and to elucidate the potential causal pathways linking trace metal homeostasis, biological aging, and cognitive health.

## Conclusion

In summary, moderate whole blood Zn levels were associated with lower odds of MCI in this occupational cohort, with the protective effect attenuating at higher concentrations and being more pronounced among individuals aged ≥ 45 years.In contrast, whole blood Cu concentrations were not significantly associated with MCI. Cross-sectional mediation analysis indicated that BA statistically accounted for approximately 28.53% of the association between Zn and MCI, highlighting the potential involvement of aging-related physiological processes. These findings underscore the importance of maintaining adequate Zn status for cognitive health in high-risk occupational populations and suggest that incorporating aging-related indicators may improve occupational health risk assessment. Longitudinal studies are warranted to clarify temporal relationships and potential causal mechanisms.

## Supplementary Information

Supplementary Material 1.

## Data Availability

The datasets underlying the results of this study can be obtained from the corresponding author upon reasonable request.
