# Supplementary material for: Zinc and copper status, biological aging, and mild cognitive impairment: cross-sectional mediation analysis in a high-risk occupational population
Source: BMC Public Health. 2026 Mar 17;26:1348. doi: 10.1186/s12889-026-27057-1 (PMC13107743; doi:10.1186/s12889-026-27057-1)
Supplement: Supplementary file 1 — Supplementary Material 1. [file 12889_2026_27057_MOESM1_ESM.docx]

| Variables | Included  (N=585) | Excluded  (N=239) | *P* |
| --- | --- | --- | --- |
| Age, $\bar{\text{x}}\text{±s}$ | 45.42$\text{±}\text{6.25}$ | 46.34$\text{±}\text{7.14}$ | 0.069 |
| Gender, n (%) |  |  | **＜0.001** |
| Male | 559 (95.6) | 201 (84.1) |  |
| Female | 26 (4.4) | 38 (15.9) |  |
| Marital status, n (%) |  |  | 0.887 |
| Married | 557 (95.3) | 229 (95.8) |  |
| Separated | 2 (0.3) | 1 (0.4) |  |
| Widowed | 16 (2.7) | 7 (3.0) |  |
| Divorced | 3 (0.5) | 1 (0.4) |  |
| Never married | 7 (1.2) | 7 (0.4) |  |
| Smoking, n (%) |  |  | 0.630 |
| No | 184 (31.4) | 80 (33.5) |  |
| Yes | 401 (68.6) | 159 (66.5) |  |
| Alcohol consumption, n (%) |  |  | 0.748 |
| No | 397 (67.9) | 155 (64.9) |  |
| Yes | 188 (32.1) | 84 (35.1) |  |
| Educational level, n (%) |  |  | **0.027** |
| Junior high school and below | 274 (46.8) | 106 (44.4) |  |
| Junior college and high school | 16 (2.7) | 16 (6.7) |  |
| Bachelor degree or above | 295 (50.5) | 117 (48.9) |  |
| Monthly income, (Yuan) |  |  | **＜0.001** |
| ＜6,000 | 189 (32.3) | 123 (51.5) |  |
| 6,000-10,000 | 296 (50.6) | 87 (36.4) |  |
| ＞10,000 | 100 (17.1) | 29 (12.1) |  |
| Diabetes, n (%) |  |  | 0.183 |
| No | 527 (90.1) | 223 (93.3) |  |
| Yes | 58 (9.9) | 16 (6.7) |  |
| BMI and Hypertension were not compared because more than 90% of excluded participants had missing data for these variables. | | | |

**Table S1.** Comparison of baseline characteristics between included and excluded participants.

| Variables | Missing（N） | Missing（%） |
| --- | --- | --- |
| Marital status | 61 | 10.3 |
| Educational level | 25 | 4.22 |
| Monthly income | 58 | 9.8 |
| Smoking | 56 | 9.46 |
| Alcohol consumption | 70 | 11.82 |
| Height | 8 | 1.35 |
| Weight | 8 | 1.35 |
| Systolic blood pressure | 8 | 1.35 |
| Diastolic blood pressure | 8 | 1.35 |
| FBG | 8 | 1.35 |
| HbA1c | 8 | 1.35 |

Table S2. Missing data and baseline characteristics of study participants.

Table S3. Correlation coefficients between candidate biomarkers and chronological age.

| Variable | Correlation coefficient | Variable | Correlation coefficient |
| --- | --- | --- | --- |
| **SBP** | **0.186** | **MCV** | **0.153** |
| DBP | 0.079 | **MCH** | **0.125** |
| PLS | 0.040 | MCHC | 0.052 |
| **FVC** | **0.112** | PLT | 0.081 |
| FEV1 | 0.033 | PLTV | 0.021 |
| **ALT** | **0.143** | PCT | 0.077 |
| **PTR** | **0.142** | PLTW | 0.023 |
| AST | 0.013 | **RBCCV** | **0.143** |
| GT | 0.009 | USG | 0.003 |
| TG | 0.056 | UPH | 0.085 |
| TC | 0.025 | **ALB** | **0.217** |
| HDL | 0.096 | ALP | 0.006 |
| LDL | 0.042 | TP | 0.055 |
| GLU | 0.033 | GLO | 0.006 |
| WBC | 0.052 | TBIL | 0.035 |
| **NEUP** | **0.102** | DBIL | 0.008 |
| **LYMP** | **0.148** | IBIL | 0.049 |
| MPNOP | 0.088 | **UA** | **0.213** |
| ACTP | 0.037 | CR | 0.010 |
| BASP | 0.047 | BUN | 0.084 |
| MWBC | 0.005 | CRP | 0.056 |
| **LYM** | **0.161** | GHB | 0.061 |
| MONO | 0.055 | **PTA** | **0.144** |
| BAS | 0.014 | **APTTL** | **0.112** |
| ACT | 0.058 | FIBL | 0.072 |
| **RBC** | **0.177** | TTL | 0.016 |
| HG | 0.087 | **PT** | **0.145** |
| HCT | 0.068 | BMI | 0.076 |
| Bold values indicate correlation coefficients with an absolute value greater than 0.1 (\|r\| > 0.1).  **Abbreviations:** SBP, systolic blood pressure; DBP, diastolic blood pressure; PLS, pulse rate; FVC, forced vital capacity; FEV1, forced expiratory volume in one second; ALT, alanine aminotransferase; PTR, prothrombin time ratio; AST, aspartate aminotransferase; GT, γ-glutamyl transferase; TG, triglycerides; TC, total cholesterol; HDL, high-density lipoprotein; LDL, low-density lipoprotein; GLU, glucose; WBC, white blood cell count; NEUP, neutrophil percentage; LYMP, lymphocyte percentage; MPNOP, monocyte percentage; ACTP, eosinophil percentage; BASP, basophil percentage; MWBC, mid-size white blood cell count; LYM, lymphocyte count; MONO, monocyte count; BAS, basophil count; ACT, eosinophil count; RBC, red blood cell count; HG, hemoglobin; HCT, hematocrit; MCV, mean corpuscular volume; MCH, mean corpuscular hemoglobin; MCHC, mean corpuscular hemoglobin concentration; PLT, platelet count; PLTV, platelet volume; PCT, plateletcrit; PLTW, platelet distribution width; RBCCV, red blood cell distribution width-coefficient of variation; USG, urine specific gravity; UPH, urine pH; ALB, albumin; ALP, alkaline phosphatase; TP, total protein; GLO, globulin; TBIL, total bilirubin; DBIL, direct bilirubin; IBIL, indirect bilirubin; UA, uric acid; CR, creatinine; BUN, blood urea nitrogen; CRP, C-reactive protein; GHB, glycosylated hemoglobin; PTA, prothrombin activity; APTTL, activated partial thromboplastin time (long); FIBL, fibrinogen; TTL, thrombin time; PT, prothrombin time; BMI, body mass index. | | | |


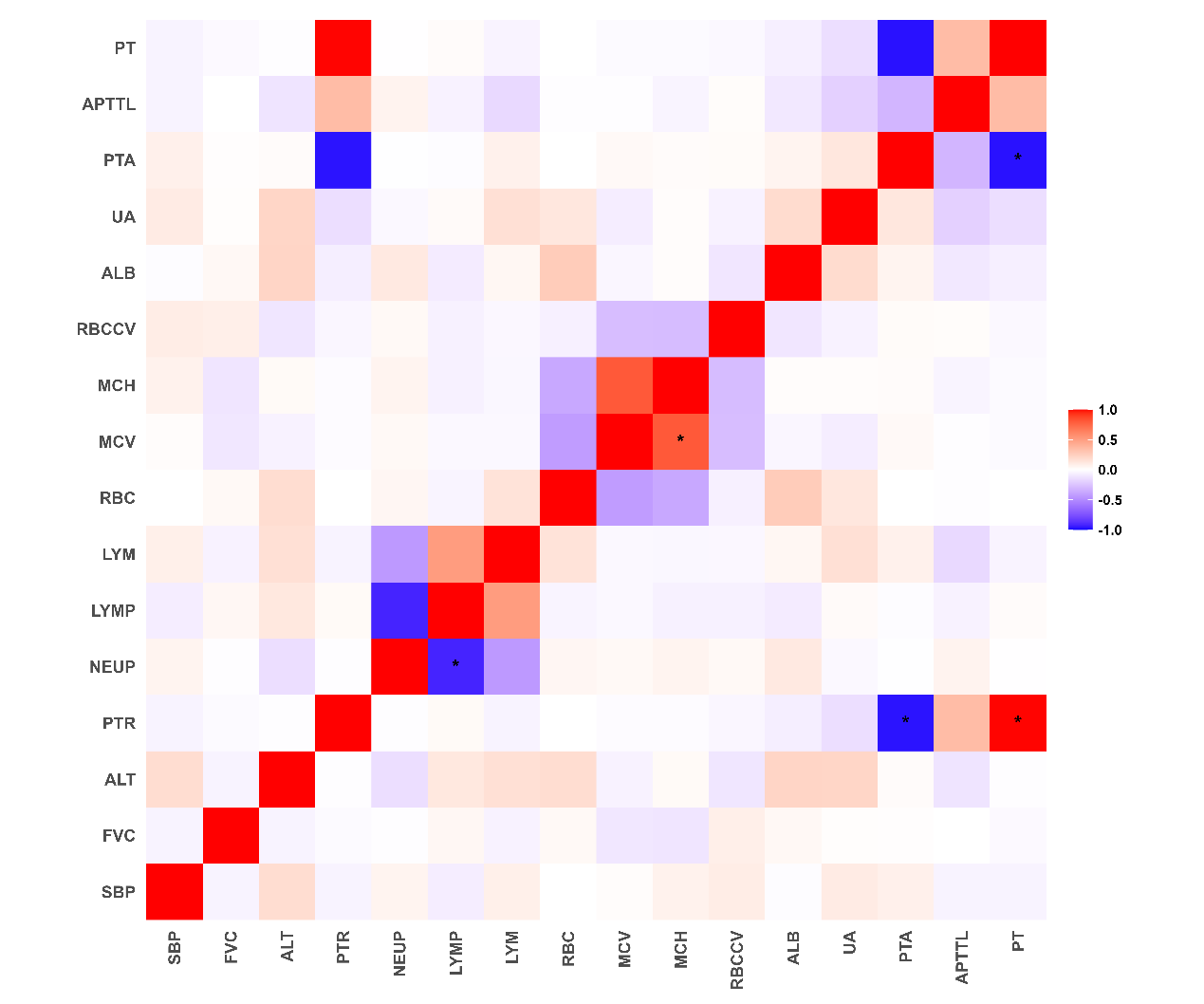
**Figure S1.** Spearman correlation matrix among selected biomarkers. An asterisk (*) indicates a strong correlation ( |r| > 0.7 ).

| Metal  Median (P_25,_ P_75_) | MCI  (N=326) | Non-MCI  (N=259) | *P* |
| --- | --- | --- | --- |
| **≤45** |  |  |  |
| Zn | 17.1 (15.2,19.0) | 17.2 (15.8,18.4) | 0.929 |
| Cu | 16.3 (14.0,18.0) | 16.6 (14.8,18.8) | 0.318 |
| **＞45** |  |  |  |
| Zn | 16.2 (14.8,18.1) | 17.1 (15.9,18.8) | **0.010** |
| Cu | 17.0 (15.0,19.0) | 16.4 (15.0,18.3) | 0.169 |

**Table S4.** Distribution of whole-blood Zn and Cu concentrations by cognitive status and age subgroup.

| Metal | Variables | Continous | Quartile 1 | Quartile 2 | Quartile 3 | Quartile 4 | *P* for trend |
| --- | --- | --- | --- | --- | --- | --- | --- |
| **≤45** (N = 287) |  | | | | | | |
| Zn | Model 1 | 1.01 (0.93,1.11) | 1(Ref.) | **0.49 (0.24,0.97)** | 0.58 (0.30,1.14) | 0.96 (0.50,1.87) | 0.087 |
|  | Model 2 | 1.02 (0.93,1.11) | 1(Ref.) | **0.50 (0.25,0.99)** | 0.60 (0.30,1.17) | 0.96 (0.50,1.87) | 0.106 |
|  | Model 3 | 1.00 (0.91.1.10) | 1(Ref.) | 0.51 (0.25,1.07) | 0.53 (0.26,1.09) | 0.92 (0.46,1.84) | 0.143 |
| Cu | Model 1 | 1.00 (0.97,1.02) | 1(Ref.) | 0.67 (0.35,1.28) | 0.92 (0.48,1.75) | 0.62 (0.32,1.20) | 0.404 |
|  | Model 2 | 1.00 (0.97,1.02) | 1(Ref.) | 0.68 (0.35,1.30) | 0.94 (0.49,1.78) | 0.64 (0.33,1.24) | 0.447 |
|  | Model 3 | 1.01 (0.98,1.03) | 1(Ref.) | 0.72 (0.36,1.46) | 1.03 (0.52,2.06) | 0.71 (0.35,1.43) | 0.604 |
| **＞45** (N = 298) |  | | | | | | |
| Zn | Model 1 | **0.90 (0.82,0.97)** | 1(Ref.) | **0.39 (0.20,0.79)** | **0.43 (0.21,0.90)** | **0.40 (0.20,0.83)** | **0.034** |
|  | Model 2 | **0.90 (0.82,0.97)** | 1(Ref.) | **0.38 (0.19,0.76)** | **0.44 (0.21,0.92)** | **0.40 (0.20,0.83)** | **0.031** |
|  | Model 3 | 0.93 (0.85,1.02) | 1(Ref.) | **0.45 (0.21,0.96)** | 0.51 (0.24,1.12) | 0.59 (0.27,1.27) | 0.199 |
| Cu | Model 1 | 1.06 (0.98,1.13) | 1(Ref.) | 0.66 (0.33,1.30) | 0.90 (0.45,1.81) | 1.51 (0.73,3.12) | 0.128 |
|  | Model 2 | 1.05 (0.98,1.12) | 1(Ref.) | 0.64 (0.32,1.26) | 0.85 (0.42,1.71) | 1.42 (0.68,2.93) | 0.148 |
|  | Model 3 | 1.06 (0.98,1.14) | 1(Ref.) | 0.64 (0.31,1.35) | 0.81 (0.38,1.73) | 1.52 (0.60,3.33) | 0.134 |
| Model 1 was unadjusted;  Model 2 was adjusted for age and gender;  Model 3 was adjusted for age, gender, smoking, alcohol consumption, educational level, and monthly income;  Bold values indicate statistical significance (p < 0.05). | | | | | | | |

**Table S5.** Odds ratios (ORs) and 95% confidence intervals (95% CIs) for MCI across quartiles of whole blood Zn and Cu concentrations in age subgroups.

**
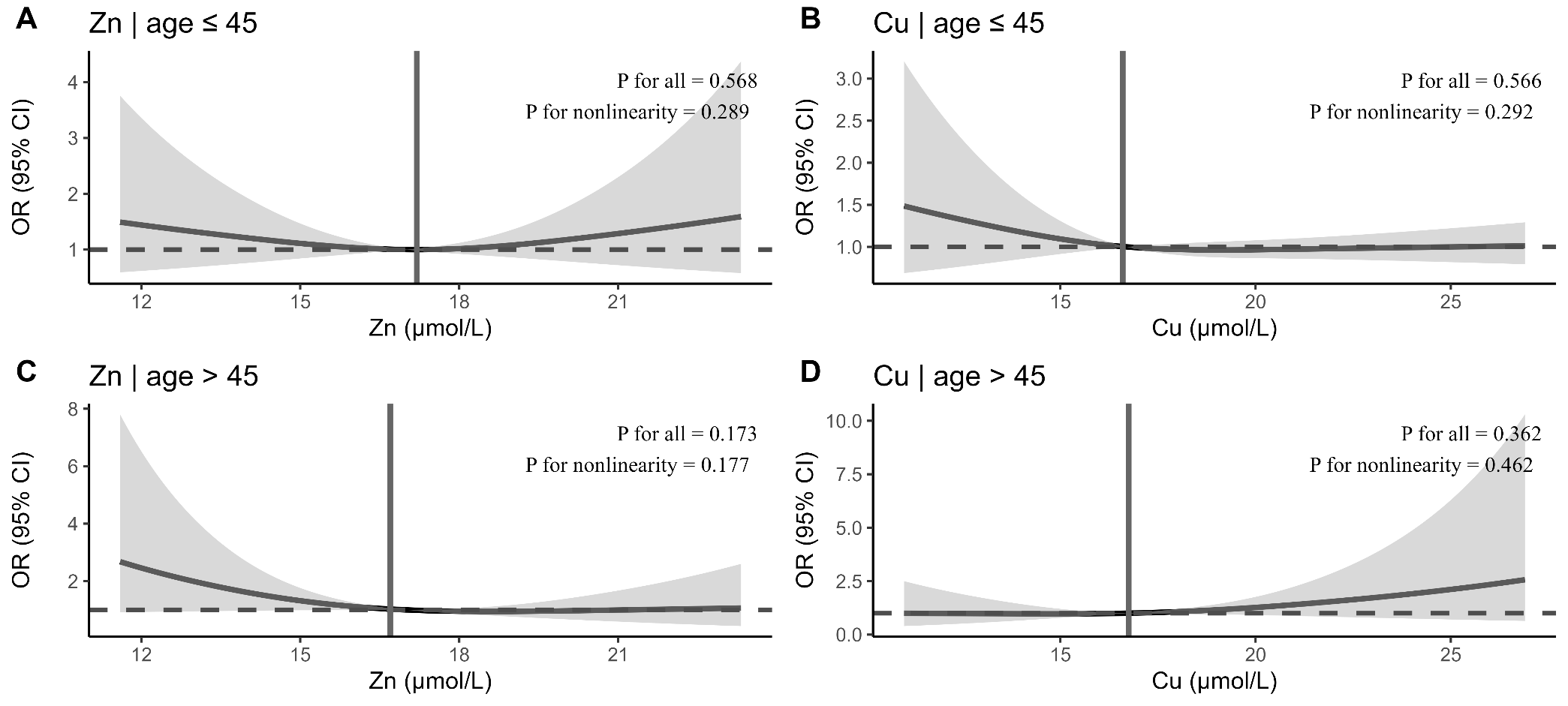
**

**Figure S2.** Dose–response relationships between whole blood zinc and copper concentrations and mild cognitive impairment (MCI), stratified by age (N = 585). (A-B) Associations among participants aged ≤ 45 years (n = 287) for Zn (A) and Cu (B); (C-D) associations among participants aged > 45 years (n = 298) for Zn (C) and Cu (D). Solid curves represent adjusted odds ratios (ORs), and shaded areas indicate 95% confidence intervals (95% CIs). All models were adjusted for age, gender, smoking, alcohol consumption, educational level, and monthly income.
